# Digital Reconstruction: A Critical Examination of the History and Adaptation of Ku Klux Klan Websites

**DOI:** 10.1177/08862605241260002

**Published:** 2024-08-09

**Authors:** Ashton Kingdon, Aaron Winter

**Affiliations:** 1 University of Southampton, UK; 2 Lancaster University, UK

**Keywords:** Ku Klux Klan, racism, whiteness, far right, online extremism, cyber crime

## Abstract

In response to the data revolution, academic research and media attention have increasingly focused on the technological adaptation and innovation displayed by the far right. The greatest attention is paid to social media and how groups and organizations are utilizing technological advancement and growth in virtual networks to increase recruitment and advance radicalization on a global scale. As with most social and political endeavors, certain technologies are in vogue and thus draw the attention of users and regulators and service providers. This creates a technological blind spot within which extremist groups frequently operate older and less well regarded technologies without the oversight that one might expect. This article examines the less well-studied traditional and official websites of the Ku Klux Klan, the most established and iconic of American far-right organizations. By incorporating non-participant observation of online spaces and thematic analysis, this research analyzes the evolution of 26 websites, from their emergence in the early 1990s to the present day. We examine the ways in which traditional printed communications and other ephemera have progressed with advances in technology, focusing on the following central elements of Klan political activism and community formation: Klan identity, organizational history, aims and objectives; technology and outreach, including online merchandise and event organization; and the constructions of whiteness and racism. The results add value and insight to comparable work by offering a unique historical insight into the ways in which the Klan have developed and made use of Web 1.0, Web 2.0, and Web3 technologies.

## Introduction

In response to the data revolution and resurgence and mainstreaming of the far right, as well as the convergence of the two, in recent years, academic research and media attention have increasingly focused on the technological adaptation and sophistication displayed by the latter (Winter, 2019; Mondon and Winter, 2020). The greatest attention has been placed on the influence and impact of Web 2.0 (Miller-Idriss, 2020; Kakavand, 2022; Leidig, 2023), and particularly how groups and organizations are utilizing technological advancements and virtual networks to increase recruitment and radicalization and disseminate propaganda in non-centralized or “top-down” ways. The importance of understanding the ways in which such technology and social media platforms help such practices is well established, but history has shown that research emphasizing single “primary” factors that affect the impact of extremist activity, fails to recognize complex multifactorial and interrelated influences, including subcultural and organizational ones. Once, considerations of organizations as primary top-down drivers of far-right activity and mobilization served as a distraction from an exploration of more bottom-up and diffuse forms and modes of activity. In the past few years, however, this has been inverted, with a shift to research focused on dispersed, “bottom-up,” and individualized “lone-actor” aspects. This has similarly diverted attention from the examination of the role of traditional organizations, and their interactions with individuals, as well as different technologies and platforms.

For this reason, this article examines the less well-studied traditional and official white supremacist websites of the Ku Klux Klan, the most iconic of American far-right organizations, and their role and function as incubators for past, present, and future far-right recruitment, organization, strategies, and activism. In a sense, we are looking not at a hidden space of hate per se, but a public arena that has been obscured by the focus on both “new” movements, trends and technologies. Our analysis of 26 websites from the early 1990s to the present day offers a unique insight into the way that the Klan’s communication, recruitment, organization, strategies, and activism have developed and progressed with advances in technology. The results of this paper center on the following central elements of Klan political activism and community formation: Klan identity, organizational history, aims and objectives; technology and outreach, including online merchandise and event organization; and the constructions of whiteness and racism.

## Literary Overview

### The Far-Right Online

The World Wide Web is a global information infrastructure that currently underpins, reflects, and influences the economic, cultural, social, and political power, interactions, and formations of a large part of the global population. It depends on a simple model of information exchange, utilizing Hypertext Transfer Protocol (HTTP) to access resources and documents that have been formatted in the Hypertext Mark-Up Language (HTML; Kingdon & Ylitalo-James, 2023). This technology has seen innovation and evolved over time from the “Web of Documents”—or read only Web (Web 1.0), through the “Web of the People”—the read-write Web (Web 2.0), to the “Web of Linked Data”—the Web for machines (Web 3.0—The Semantic Web), and subsequently Web3—a series of open-source and interconnected decentralized applications powered by blockchain computing architecture (please see Hawes, 2023; O’Hara & Hall, 2021; for an in-depth description of the terminology).

As the Web evolved, so too did our economic, cultural, social, and political interactions and formations, including extremism, with many groups and organizations utilizing technological advancements and ever-expanding virtual networks to increase recruitment and advance radicalization on a global scale (Conway, 2017). It is widely accepted that the Web has become a virtual “forecourt” for the promotion of far-right ideology and activism, and influence on individuals receptive to recruitment and radicalization and their targets and victims (Daniels, 2009; Kingdon, 2021; Scrivens, 2021; Zempi & Awan, 2016). Analysis of the far right’s use of the Web to recruit and radicalize, as well as spread their ideas, has generally focused on the content featured on websites (Back, 2002; Blazak, 2001; Brown, 2009; Levin, 2002; Perry & Olsson, 2009), and Web forums (Bowman-Grieve, 2009; Caren et al., 2012; De Koster & Houtman, 2008; Lokmanoglu & Veilleux-Lepage, 2020), but less so on the history and operation of these for the movement, organization, and wider far-right and racist systems (see Daniels, 2009; Winter, 2019).

Studies into the influence of far-right social networks and their underlying technical structures indicate that the power of these networks stems from their operations and technical function, rather than from individuals using platforms to attract potential recruits (Caiani & Parenti, 2009; Holt et al, 2018; Meddaugh & Kay, 2009). Research has also demonstrated the ability for the far right to utilize the internet to aid in the construction of identity for organizations, as well as influence their mobilization, organizational contacts, and action strategies (Caiani & Parenti, 2013). Although literature has shown that online activity is universal, undertaken by individuals, and informal and formal networks, the focus of this article is on the websites and activity of organizations.

To date, a large amount of research on the far right, and that classified as extremism more generally, has concentrated on the present, exploring the use of new technological developments and trends (DeCook & Forestal, 2022; Jasser et al., 2021; Kingdon & Krause, 2021). However, the focus on extremist websites in these earlier studies has given way in recent years, (together with work on more traditional non-Web based communication), to a focus on social media and radicalization. While this may be a reflection of growing trends on the far right, and society more broadly, it now obscures the role that far-right organizations and websites play in communicating and shaping the extremist ecosystem—both online and offline—by overlooking the specificities of organizational communication, activity, and platforms. This also has the effect of disarticulating the longer history and strategies of official organizational communication (e.g., print ephemera) and presenting contemporary digital extremism as both more diffuse, and, as a pseudo “democratized,” bottom-up project. Instead, both facets must be understood as working together to shape the far right, their identity and activism. For this reason, this article will now turn to the past, and review the history of the Ku Klux Klan as an organization, how it operated and communicated its ideas, and how they related to social, political, cultural, and technological change.

### The Ku Klux Klan: From Night Riding to Online Posting (A Short History)

The history of the Ku Klux Klan is organized into eras, of which there have been five, three formal and established eras (1–3) and two partially overlapping ones that were less concrete and centralized (4–5). In each era, the Klan emerged and mobilized in response to social and political developments, such as Reconstruction and Civil Rights. Such activity was, ultimately about the changing relationship between race, state, and nation, and the defense or preservation of whiteness in “America” as the Klan represented it. This informed and was articulated in new adaptations of their branding and ideology. In each era of mobilization, the Klan used new strategies and tactics including physical violence, public rallies and forms of communication used to propagandize, recruit, and sell their identity, ideas, and merchandise, such as manifestos, periodicals, and pamphlets (Winter, 2018). As the Web developed, the Klan expanded their communicative strategies to include websites and social media platforms, alongside these longer standing and more traditional mediums.

The Klan was founded by Nathan Bedford Forrest in Pulaski, Tennessee in 1865 as a social club for veterans of the Confederate army and became an explicitly political organization in 1868 (Winter, 2018). It provided an outlet and organized representation for white disenchantment, resentment, and opposition to the abolition of slavery, reconstruction, and “northern carpetbaggers” (Newton, 2014). The first-era Klan was organized according to local dens, but was unified as a southern movement in 1868, ending in 1873 following federal intervention (Chalmers, 2007). This period was immortalized in Thomas Dixon’s (1905) book *The Clansman* (and the trilogy it belonged to) and D.W. Griffith’s 1915 film *Birth of a Nation* based on it.

In 1915, the same year as the film’s release, “Colonel” William Joseph Simmons and five others established the second-era Klan at Georgia’s Stone Mountain. During this period, the organization was quite active and prolific in writing and publishing, including *Searchlight*, *Imperial Night-Hawk*, and *The Kourier*. Despite its southern roots, and continuing organization around state-based Knights klaverns, the second-era Klan was attractive nationally and in the mainstream because of its patriotic appeal (Gordon, 2017). Following World War 1, the Klan mobilized around anti-immigrant nativism and 100% Americanism, and against Jews, Catholics, and Asians, as well as Black Americans and ‘‘communists’’ (Winter, 2018). Mainstream power came when President Woodrow Wilson expressed support and Congress passed the Klan-supported 1924 Immigration Act (Johnson-Reed Act). This Klan would decline in the second half of the 1920s partly due to criminal charges for rape and murder against Grand Dragon D.C. Stephenson. The Klan would be overtaken by fascists in terms of position and prominance the 1930s.

The third-era Klan came into force in the 1950s in opposition to desegregation and continued to grow in opposition to Civil Rights in the 1960s (Ridgeway, 1990). The third-era represented a return to the organization’s southern, Confederate roots, being split across three factions: *The Original Knights of the Ku Klux Klan*, *The White Knights of the Ku Klux Klan*, and *United Klans of America*. While the Klan of this era had mainstream support and used legitimate political strategies, it also returned to the violence and intimidation of the first-era. The next stage of Klan development and adaptation in the late 1970s and 1980s, which can be described as the fourth-era, was largely defined by a mainstreaming and media strategy led by David Duke, Grand Wizard from 1975 to 1980. Duke ran for elected office, including the Presidential nomination in 1988 and a successful run for the Louisiana House of Representatives in 1989, and formed the National Association for the Advancement of White People. Tom Metzger, of the California Knights, replaced Duke as Grand Dragon, ran for elected office, ran the public access show, *The World as We See It*, later renamed *Race and Reason* (Winter, 2018), and appeared on mainstream TV talk shows. He also formed the less mainstream and more extreme and insurgent *White Aryan Resistance*.

The fifth-era began in 1981, overlapping with the fourth, and lasted until the mid-1990s. It was represented by the paramilitarization of more radical Klans who rejected their predecessor’s and Duke’s mainstream aspirations and strategies. They included Louis Beam Jr’s *Texas Emergency Reserve* (Texas Knights) and Frazier Glenn Miller’s *White Patriot Party* (North Carolina Knights). This was most clearly expressed by Beam himself in the Spring 1984 *Inter-Klan Newsletter & Survival Alert*: “*where ballots fail, bullets will prevail*” (Ridgeway, 1990, p. 87; Winter, 2019). The Klan would be largely surpassed in this era by the more radical, fascist and explicitly antisemitic, National Alliance and Christian Identity affiliated organizations, such as Posse Comitatus, The Order, and Aryan Nations. The latter was led by former California Klansman Richard Butler who also attempted to unify the diverse strands and factions of the far right through his annual Aryan World Congress (Winter, 2018).

While most of these organizations continued with printed ephemera, including Beam’s (1983) *Essays of a Klansman*, the other major innovation for the Klan in this period was going online. In the Spring 1984 issue of the *Inter-Klan Newsletter and Survival Alert*, Beam also announced *Aryan Nations Liberty Net* or *Aryan Nations/Ku Klux Klan Computer Net*, the first white supremacist online system and bulletin board (Berlet, 2008; Winter, 2019). In the article “*Computers and the American Patriot*,” Beam wrote, “*American know-how has provided the technology which will allow those who love this country to save it from an ill-deserved fate*” (Berlet, 2008). In the 1980s and 1990s, far-right computer networks were often predicated on escaping government surveillance (Winter, 2019), and mobilizing allegedly persecuted or isolated white activist communities across long distances (Back et al., 1998). For this reason, the Klan’s early digital media was designed for existing members, and often included access codes as a way to exclude infiltrators and prevent surveillance. Soon after Beam’s announcement, Tom Metzger announced the *W.A.R. Computer Terminal* and established *The Insurgent Network*. Metzger described the Insurgent as “*a NETWORK of highly motivated White Racists. Each person is an individual leader in his or her own right. THE INSURGENT promotes the Lone-Wolf tactical concept. Made up of individuals and small cells*” (in Daniels, 2009, p. 103). According to Daniels (2009, pp. 103–104), this notion of “leaderless resistance” was introduced by Beam as well around the same time and would become popular and influential across the wider movement in the 1990s. In fact, when Beam revisited it in 1992 at the Christian Identity Rocky Mountain Rendezvous, it played a role in the mobilization of the anti-government militia movement, as well as far-right plots and attacks. In this way, we can see the way ideologues not only employed technology for traditional propaganda and recruitment, but used it to inform and develop strategies that would dominate the era and succeeding years.

Thomas Robb, National Director of the Knights of the Ku Klux Klan, Christian Identity Minister, and editor of *The Torch*, purchased the domains *kkk.com* and *kkk.biz* in the early 1990s. For Robb, there was continuity between print and the Web as he developed a cut/paste technique whereby he uploaded print articles directly from *The Torch* to his websites (Daniels, 2009). Therefore, despite digitization, *The Torch* remained fairly traditional and did not make full use of the technology available. Although it is at this time, as the Web was becoming more popular and accessible, that more public-facing engagement became apparent, as the websites were utilized for wider recruitment, outreach, and mobilization. The most notable example of this was the 1995 development of former Klansman Don Black’s *Stormfront* in 1995. Yet, Robb’s websites remained active for decades and form part of the basis for our data sample. While the Klan underwent a significant decline in the first decade of the 2000s, it experienced increase attention following the 2008 election of Barack Obama, and in 2015, amidst the Presidential campaign of Donald Trump, where Klan chapters grew from 72 to 190 (SPLC, 2016). This was part of a wider revival of the far right that the Klan was a small part of and largely overshadowed by the more popular and diffuse Alt-Right groups who have used both social and traditional, as well as mainstream, media in new and successful ways (Winter, 2019). Although bereft of funds and able leadership, and with only scattered membership, the Klan nonetheless remains a potent historic symbol of racist terrorism and violence, which individual members still commit. The Klan also maintain a fragmented online presence on other platforms and networks, and through their own official websites, which will be examined and discussed in the results section of this article.

## Methodology

This research employed the epistemological position of interpretivism, utilizing qualitative research methods and non-probability data-gathering techniques to provide a comprehensive understanding of the historical and technological adaptation and evolution of Ku Klux Klan websites. It does so by examining its content based around a number of themes and areas over a period of social, political, and organizational change and technological development to understand the relationship between these. As interpretivist methods produce data that helps uncover meanings and insight, it was the most beneficial for this study. Methodologically, this research incorporated non-participation observation of online spaces and thematic analysis, taking an inductive approach, to allow for potential themes to emerge naturally from the data that was collected (Hennink et al., 2011). While research has previously investigated the activity and community formation present on Klan websites, these have either been limited to analyses of individual platforms (Selepak & Sutherland, 2012; Spicer, 2018; Waltman, 2003), or are snapshots of particular moments in time (Bostdorff, 2004; Gerstenfeld et al., 2003; Schmitz, 2016). This research adds value and insight to comparable work by offering a unique historical insight into the development and evolution of Klan websites, detailing how they have progressed and made use of Web 1.0, Web 2.0, and Web3 technologies.

### Data Sampling

Due to the size, dimensions, and shifting dynamics of the Web, one of the greatest challenges to web-bases research is determining an appropriate population or content from which to draw a representative sample (Schafer, 2002). Therefore, non-probability sampling techniques were utilized to collect the most relevant data for this study. The other main rationale for this choice of sampling is that, as gaining access to extremist content can be difficult, information needs to be gathered from locations that are available (Litchman, 2014). Initially, convenience sampling was used, and data selected opportunistically based on its accessibility. It is the most suitable technique for research such as this, where members of the sample population, or the data is difficult to locate (Bryman, 2012). This research also employed purposive sampling, with the researcher collecting data based on their prior understanding of the research field and population. Due to the sensitive nature of this research, no structured inclusion criteria is included in the sampling strategy, although the exclusion criteria included any information shared by private accounts, or closed group conversations.

### Data Collection

This research involved non-participant observation of online spaces and the collection of texts publicly available on 26 official Klan websites that had a temporal coverage significant enough that changes could be tracked over time (Table 1). The choice to collect this sample size centered on the fact that as data was collected manually, this would provide an appropriate amount of data so that trends could be tracked over time. Although computational methods of data collection have proven successful when analyzing white supremacist websites and forums, particularly via the use of linguistic inquiry and word count analysis^1^ (Crabill, 2008; Figea et al, 2016; Weeda et al, 2022), this study was not solely concerned with content, but also the methods, technologies, and approaches employed by the users, creators, and hosts of the websites. Moreover, many Web-scraping technologies are employed to investigate bulk data via advance networks and textual analysis methods; as our study was inductive in nature, and predominantly collected via archives, this would not have been the most effective method.

**Table 1.** List of Websites Used in Analysis Including Names, URLs, and Temporal Coverage.

Name	URL	Temporal Coverage	Data Collection Period
Association of Klans, Knights of the Ku Klux Klan	Associationofklanskkkk.weebly.com	2009–2019	03-2019 to 08-2022
Brotherhood of Klans	Knightskkk.com	2010–2018	03-2019 to 08-2022
Church of the National Knights of the Ku Klux Klan	Cnkkkk.net	2011–2019	03-2019 to 08-2022
Church of the United Realms of American Knights of the Ku Klux Klan	Curakkkk.com	2008–2019	03-2019 to 08-2022
Dixie Rangers Louisiana of the Ku Klux Klan	Dixierangerskkk.com	2005–2019	03-2019 to 08-2022
Fraternal White Knights of the Ku Klux Klan	Knights311.org	2009–2019	03-2019 to 08-2022
Imperial Klans of America Knights of the Ku Klux Klan	Kkkk.net/texas	2005–2007	03-2019 to 08-2022
International Keystone Knights	Ikkkkk.org	2008–2021	03-2019 to 08-2022
KKK—The Ku Klux Klan	Kkklan.com	1998–2022	03-2019 to 08-2022
Knight Riders Knights of the Ku Klux Klan	Knightriderskkkk.com	2009–2013	03-2019 to 08-2022
Knights of the Ku Klux Klan Party	Kkk.bz	2004–2022	03-2019 to 08-2022
Knights Party Veterans League	Knightspartyveteransleague.com	2008–2022	03-2019 to 08-2022
Ku Klux Klan	Kkk.com	1998–2020	03-2019 to 08-2022
Mississippi White Knights	Mwkkkk.com	2004–2021	03-2019 to 08-2022
Newport Tennessee Knights Party	Newporttennessee.net	2003–2011	03-2019 to 08-2022
North Carolina Knights of the Ku Klux Klan	Worldknights.org	2004–2011	03-2019 to 08-2022
Southern Alliance of Klans	Sakkkk.com	2008–2021	03-2019 to 08-2022
Texas Rebel Knights of the Ku Klux Klan	Texasrebelknightskkk.com	2010–2022	03-2019 to 08-2022
The Brotherhood of Klans knights of the KKK	Bok33.org	2004–2014	03-2019 to 08-2022
The Knights	Arkpower-light.com	2003–2015	03-2019 to 08-2022
The Ku Klux Klan LLC	Kukluxklan.bz	2002–2022	03-2019 to 08-2022
Traditionalist American Knights of the Ku Klux Klan	Traditionalistamericanknights.com	2009–2022	03-2019 to 08-2022
True Knights of the Ku Klux Klan	Kkk.net	1999–2019	03-2019 to 08-2022
United Northern and Southern Knights of the Ku Klux Klan	Unskkkk.com	2005–2021	03-2019 to 08-2022
United White Knights of the Ku Klux Klan	Uwkkkk.com	2008–2022	03-2019 to 08-2022
White Camelia Knights of the Ku Klux Klan	Wckkkk.org	2004–2022	03-2019 to 08-2022

The substantial list of websites that were chosen for analysis was partly drawn from Selepak’s (2011) doctoral thesis “*White Hoods and Keyboards: An Examination of Ku Klux Klan websites*.” Additional websites were identified by one of the researchers during their data collection process through their knowledge of the research population. Eighteen of the twenty-six websites analyzed were no longer live and thus accessed using the Internet Archive “The Wayback Machine.” This tool was also used to analyze all of the websites due to the fact it provided an accurate picture of how content had evolved over time through its in-built “changes” feature, which allows for comparisons of different versions of the same archived page. The same data was collected across all of the websites, including the homepage, membership applications, historical background, events pages, merchandising, open community forums, news outlets, and any external links (e.g., to merchandise sale platforms, radio stations, television shows, or other websites). The inductive analysis compliments the nature of this study, as it made no attempt to code the text into a preexisting framework and thus enabled narratives to emerge organically throughout.

### Data Analysis

The data chosen included that focused on three central elements of Klan political activism, community formation, and communication: Klan organization history and technological innovation; outreach, including recruitment, events, and merchandising; and constructions of whiteness and racism. The choice of these three areas was based on how they enabled analysis of the organization’s racial and political discourses and external engagement, and to map changes to and across websites over time and in conjunction with emergent technologies. Although content analysis would have been a feasible option for examination due to its reliable, objective, systematic, and quantitative breakdown of content, it is most suited to outlining the obvious and self-evident features of data, and not the hidden dimensions (Berelson, 1952). This research instead employed thematic analysis, as the most appropriate for identifying and analyzing patterns within the data collected, in an inductive way, from the ground up. The technique utilized bore resemblance to Braun and Clarke’s (2006) “Six Phases of Thematic Analysis,” although, in this research, the examination was recursive rather than linear. The process was implemented systematically, with each item of data examined individually, and care being taken to ensure the interpretation of later items were not influenced by earlier analyses. An outline of the codes and themes generated from our analysis can be seen in Table 2.

**Table 2.** Themes and Codes Used in Analysis.

Theme	Code
The Klan as an Organization	Ku Klux Klan
	Confederacy
	Heritage
	Brotherhood
	Fraternity
	Community
	Lost Cause
	Civil War
	Religion
	Family Values
	Social Class
	Reconstruction
	Links to other Groups
Technological Innovation	Web 1.0
	Web 2.0
	Web 3
	Hyperlinks
	Graphics
	Media Communication
	Public Forums
	Private Forums
	Aesthetics
	Multimedia
	Cybersecurity
	Communication for Members
Outreach	Rallies/Marches
	Mass Gatherings
	Anniversary Events
	Conferences
	Propaganda
	Online/Offline Mobilization
Whiteness/Racism	Illiberal Racism
	Liberal Racism
	Whiteness
	Racist Symbols
	Claims to not be Racist
	Christian Nationalism
	Victimization
	Great Replacement Theory
	Culture Wars
	International News Articles
	National News Articles
	Cultural Nationalism
	White Supremacy
	White Nationalism
	Alt-Right
	Mainstreaming
Merchandise	Evolving Revenue Streams
	Adjacent Platforms
	Clothing Apparel
	Klan Regalia
	Historical Memorabilia
	Homeware
	Translation Services
	Currency Converters

### Ethical Considerations

This research received full ethical approval (ERGO/47657). The ethical implications that come from researching extremism and radicalization, particularly in relation to researcher safety, mental health, cybersecurity, and institutional responsibility have been carefully considered for this study and extensively discussed by the researchers in the following publications (Ashe et al., 2021; Mattheis & Kingdon, 2021; Winter, 2024). For this study, the principal ethical considerations were how to address concerns regarding anonymity and whether it would be ethically necessary to seek informed consent due to the fact this data was collected from websites, which raised questions as to whether such information would be considered “private” or “public” (Kingdon, 2021). We recognize that research conducted on websites should not be justified as ethical solely on the basis that it is in the public domain. Additionally, there are certain scenarios in which it would be necessary to seek unambiguous informed consent, such as accessing data that is specifically private, or including data from the accounts of individuals who are under the age of 18. Consequently, in this research, data was collected solely from accounts in the public domain, disseminated by the organizations themselves, and not individual users. Registration was not required to access the content, and data could thus be retrieved without interactions with members or other users (Sugiura et al, 2016). This obviated the need to get informed consent and circumvented other ethical issues such as the researcher clearly defining their intent to avoid deception, such as the creation of false identities or “sock puppet” accounts (Kingdon, 2021). Likewise, no information was collected from profiles in which users publicly stated they were younger than 18 (although user age may be almost impossible to ascertain), if it is not specified. In compliance with the general data protection regulations, this study also exercised particular vigilance in protecting the privacy of the content creators, and, although it was the organizations were seeking to communicate with a wider public audience, all data used was strictly anonymized and any identifiable information struck from analysis.

## Results

### The Klan as an Organization

The rhetoric, organizational rationale, and mobilizing imperative of the Klan is always predicated on the promotion and representation of whiteness and Christianity in the United States, and the relationship between these against perceived threats politically, legally, culturally, socially, economically, and demographically, most notably from other racialized groups and their alleged power and interests. But it does so in diverse and contrasting ways from Confederate “lost cause” constructions of whiteness, to European civilizational, nativist and white working-class ones.

Each website analyzed contained a detailed section dedicated to representing the history, rationale, operating logic, and aims of the organization. These centered on the definition and defense of whiteness, Christianity and America, as well as individual definitions of themselves. For example, *The Brotherhood of Klans* states: “*After the cannon fell silent and peace descended upon the battlefields of the Civil War, there came an infamous chapter of American history called “The Reconstruction.” From this era, an abyss of human misery and despair, there arose like the morning sun the Ku Klux Klan*.” Similarly, the *Imperial Klans of America* details in its mission statement: “*Keeping alive the true ideas, rituals, and beliefs of the original Ku Klux Klan for the 21st century*.” A significant code throughout analysis, and one embraced by this website, was the glorification of the first-era Klan, not just in terms of history, but its evolving organizational structure: “*For over a year after its founding in Tennessee, the Ku Klux Klan did not have a grand wizard or any real national leader. It started as a local group that spread like wildfire, the Southern men had become fed up with the Federal government and oppression of Reconstruction, and the time was ripe for clandestine armed resistance*.” While these are explicit references to the legacy of the first-era-Klan, other websites construct different and even divergent organizational identities, narratives and approaches.

Two primary organizational identities and narratives emerged from the coding process. Firstly, the positioning and portrayal of the Klan as a fraternal organization, or “brotherhood” as demonstrated by *The True Knights of the Ku Klux Klan* who state “*The Movement is designed to create a real brotherhood among men who are akin in race, belief, spirit, character, interest, and purpose. Our teachings indicate very clearly the attitude and conduct that make for real expression of brotherhood, or the practice of ‘Klannishness*’.” Secondly, is the idea that the Klan is a more radical and extremist insurgent organization engaged, in some cases, in armed paramilitary and potentially violent preparation and planning in defense of the white race. This portrayal can be seen on the homepage of the *North Carolina Knights of the Ku Klux Klan* who quote directly from Tom Metzger’s *The Insurgent Network*: “*We are highly motivated white racists, we are a leaderless resistance, made of independent individuals, and a world-wide insurgency of white European racialists*.”

In a similar vein to Bostdorff (2004), this study also found that the community-building rhetoric offered by the Web enables the Klan to reach a wide audience at little cost, and thus attract individuals who desire a sense of community. Our analysis found that some websites advocate for cooperation with wider Klans, far-right movements, and white communities, while others are more restricted racially and/or ideologically. This indicates both the urgency of the perceived crisis for white Americans and their ideological position, as well as factionalism. For example, the *Brotherhood of Klans* considers itself as a more broad-based movement stating: “*We are a fraternal, patriotic, organization promoting the ideas and ideals of Western Christian Civilisation. We shall strive to work in unity and cooperation with other Klan groups that share our same philosophies, disciplines, and traditional values in order to preserve and promote our race, heritage, and faith. We seek for membership only those individuals who display the qualities of loyalty, dedication, and the unselfish desire to secure the existence of our race and a future for white children*.” In contrast, the *Knight Riders Knights of the Ku Klux Klan* is stricter, but also opposes the more extreme and non-traditional ideological elements that became dominant in the 1980s and 1990s, and a feature of Christian Identity Congresses, stating *“[o]ur staunch stand against Neo Nazi’s, Skinheads and White Nationalists from participating at our rallies or public events has caused a whirlwind of comments on various websites. We believe in the protection and advancement of the white race, but we realise there are white people who are traitorous and undesirable among us, therefore we protect only those who are members of the Ku Klux Klan. Just because your’re white doesn’t mean we trust you!*” Similarly, the *Imperial Klans of America* also state “*We do not associate with the white nationalist party, the neo-Nazis or the skin head movements. Although they are working for white heritage and the white race, their ideologies are much different from the traditional Klans. We want to preserve the US constitution in its original form, the war our forefathers intended*.” The contrast in organizational messages enables the Klan to appeal to more people that might otherwise feel uncomfortable with more extreme ideologies, movements and subcultures.

A key finding was that some Klan sites utilized the Web 1.0 practice of direct hyperlinks to connect to other Klan sites, creating a mesh of interrelated and contextually bound resources, which indicates that these groups have an organizational or working relationship with one another. Most notably the following websites: *Georgia Knight Riders; International Keystone Knights; Mississippi White Knights; United White Knights of Texas; United White Knights of Louisiana; Texas Real Knights; Traditional Christian Knights; National Aryan Knights; Oklahoma Klan’s Invisible Empire; Great Tennessee Knights*; and *Tennessee Knight Riders* are all linked together under the “Southern Alliance of Klans.” This demonstrates collusion between the groups, provides a common identity, and indicates ideological similarity and overlap between different factions.

Notably, Thomas Robb’s *Knights Party* website has the same content and styling as the following organizations: *Ku Klux Klan; Newport Tennessee Knights; Knights of the Ku Klux Klan*. These websites, despite having different Uniform Resource Locators present with the same identity, suggesting that several linked organizations use the same template and structure for their websites, which produces familiarity and reduces overheads. *The Knights Party Veteran League*, has a different layout, yet contains the same information verbatim, demonstrating that someone has mirrored the *Knights Party* website, but changed the cascading style sheets. It could be that there are different organizations, copying content directly from one another, or that it is the same webmaster using different templates. It may also suggest that organizations are working together, yet some do not have the technical capabilities to run themselves. Nevertheless, the fact this is occurring means these organizations are behaving in the way we would expect in terms of knowledge exchange. Moreover, 12 of the 26 websites contained direct hyperlinks to the landing pages of external white supremacist websites including: *White Aryan Resistance; Stormfront; The Insurgent; Aryan Resistance Movement’ Blood and Honour*. This implies the same function as above, yet provides more variety of content for their audiences. These findings show that websites are not just ideologically linked, but also share recruitment tactics, thus demonstrating a network of communication and multidirectional inspiration.

### Klan Outreach: Events and Merchandise

According to Selepak and Sutherland (2012), Klan organizations took steps to rebrand and mainstream their image similar to that of white, religious, and political conservatives. Our study found similar engagement via Klan outreach strategies. Most notably, the Klan engaged in outreach through organized events such as rallies, marches, and mass gatherings. Seven of the websites analyzed contained information about events for local chapters, including anniversary celebrations, anti-immigration rallies, marches in protest of job losses, BBQs, cross-lighting ceremonies, and the erection of crosses on public property, as well as pilgrimage tours of former plantations. From 1999 to 2011, it was commonplace for future events to be advertised on all websites, which often coalesced with certain racialized narratives. For example, *The Church of the National Knights of the Ku Klux Klan* called upon the “*Aryan race to light a candle in remembrance of all those who had fallen in the fight against Islamic terrorism*.” Moreover, the *Brotherhood of Klans*, which seems more focused on Black Muslims in America when discussing terrorism, advertised several rallies against “*Black Gang Terrorism*” and *The Dixie Rangers Louisiana of the Ku Klux Klan* had marches and parades in homage to “*White Southern Heritage*.” This shows that the Klan utilized outreach strategies designed to increase ideological polarization and aid the development of networks that not only provide an outlet for the online communication of grievances, but also lead to offline mobilization.

In addition to advertising events for local chapters, national events were also promoted. Notably, the *Knights of the Ku Klux Klan’s* annual “Faith and Freedom Conference” and “European American Heritage Festival,” capitalize on “Old South nostalgia” to promote the supremacy of the white Christian race. The same website also endorsed the annual “White Christian Heritage Festival,” which was described as a family event with games for children, cultural exhibits, face painting and craft making. The festival is strategically placed in the town of Pulaski, Tennessee, symbolic to the Klan for being the location in which it was originally founded in 1865. Additionally, different Klan sites used events as outreach to connect their ideas or practices with other organizations, as well as the general public. For example, *The Imperial Klans for America* advertised “Nordic Fest”—an annual white power rally and music festival held in conjunction with the neo-Nazi skinhead group Blood and Honor. This type of outreach takes an educational component in relation to the propagation of ideas. In this way, the strategy is a two-way street in which the events are framed as “engagement,” rather than propaganda. This approach is directly linked to the Klan’s mission, which bolsters the narrative that people want to partake in these events to be a part of and engage with, an exclusively white organization that opposes immigration, homosexuality, and race-mixing. It is important to note that after 2012, no websites had any future events advertised and would only document past activities, signaling that activity was now much more online, which returns us to the earlier issues of group and intra-Klan relations. A feature of most collective events is merchandising, including that which marks out identity and affiliation and helps raise funds for their operation and cause.

The far right has long engaged in merchandising to create group identity and affiliation markers, as well as raise revenue. In digital contexts specifically, “merchandising” is increasingly important because the entanglement of narrative and product consumption has become the essential function of digital and social media. Here, influence is mobilized to create informational “products” such as podcasts, vlogs, and streaming radio shows (Mattheis & Kingdon, 2023). Six of the websites analyzed sold merchandise directly to Klan members and the general public. These manifest in different ways, for example the *Mississippi White Knights* have a “specialty items” section and the *Traditionalist American Knights* have a “KKK Store.” Thirteen websites had links to ideologically adjacent sites—websites selling products expressly unaffiliated with the organization, but ideologically supportive and thus white supremacist in their orientation. For example, *The Ku Klux Klan* website had a hyperlink to a website titled “*Christian Books and Things*” which sold written content relating to British Israelism, biblical prophecies, and anti-immigration and *The Empire Knights of the Ku Klux Klan* were linked to “Akia Wear” a site specifically for Klan members that sold clothing items. *The Knights Party*, had a specific section on its website dedicated to southern and Confederate merchandise titled “*The White Heritage Store*.” Only three of the websites hosted links to Klan regalia (the white robes and hood; patches etc.) and included in analysis was a website titled the *Ku Klux Klan: Ku Klos Knights* which specifically sells Klan supplies including Klorans, manuals, patches, baldrics, office patches, and Klan and Confederate flags under the title “*Everything a Klansman or Klaven Needs*.”

Common types of merchandise occurring across all sites include the following: artifacts from the original first-era Klan (leaflets, flyers, advertising materials); historical books/booklets (the original pre-script of the first-era Klan, the first decree of the Klan’s Constitution and Laws); memorabilia (calling cards, postcards, posters, window/bumper stickers); conspiratorial literature (Holocaust Denial, Zionist Occupied Government [ZOG] and other antisemitic theories, British Israelism, Christian Identity); clothing apparel (T-Shirts, shorts, bathing suits, hats, jewelry, pins, badges, watches, belt buckles, keychains, and patches); food and homeware items (plates, cutlery, aprons, oven gloves, utensils, stationary, mousepads, aprons, playing cards, and makeup). Importantly, much of the clothing apparel and homeware featured the Confederate battle flag, or traditional symbols to fascism, as well as Nordic. The diversity of material, iconography and sources of merchandise we can see across, as well as within, sites from traditional Klan to Identity and Odinist, and from Confederate to national to white European civilization level, is of importance. In many ways it signals and replicates ideological lines and recruitment strategies discussed earlier. Ideological fidelity and recruitment strategy may not be the determining factor though, as it may be linked to the space provided to populate sites with increasing volumes of potentially profitable merchandise in ways traditional printed material did not. So, the digital commodification of hate is big business for both extremists and the online platforms that host their products. Recommender and browse features have a significant influence on the range of content to which audiences are exposed, and, because such content is not restricted to clandestine areas of the Dark Web, or encrypted platforms, it instinctively appears more legitimate (Mattheis & Kingdon, 2023). This leads us to our analysis of the ways in which the Klan has capitalized on technology to aid recruitment and the advancement of their narratives.

### Technological Innovation

While technology, as a topic and influence, runs through all the data and sections due to the nature of this being online communication, we feel that because the organizations, groups, and websites discuss it differently, it should be treated as a discreet section. The technology afforded by the Web allows for the transformation of information easily, reduces friction, and lowers barriers to exchange information. From the 1990s to the early 2000s, we observe that the Klan utilized Web 1.0 technologies in the traditional creator-publisher format, with websites designed to serve as online bulletin systems, host propaganda that was primarily cut/pasted from printed communication and advertise offline events and networking opportunities.

In the mid-2000s, there was a clear shift in the technological affordances of the websites, as the Klan adopted Web 2.0 technologies and more integration was offered. In addition to having static websites, the majority of organizations provided a number of additional services including links to social media, chatrooms, radio, television, and Web forms for direct communication. We also see writings from non-affiliated far-right ideologues, such as Jerome Corsi, Pat Buchanan and Rush Limbaugh with the latter’s show being advertised on the *Knights Party* website in the early to mid-1990s. The site also posted mainstream media articles from various regions, including the *New York Daily News*, *Oregonian*, *Chicago Tribune*, *Atlanta Journal-Constitution*, and more local, city and state ones, as well as international including *Ireland Online* and *Manchester Evening News*. We see direct engagement with these articles from webmasters, recognized idealogues, and Klan members and supporters demonstrating engagement with distinct narratives that will be discussed below. Although seemingly mundane, we also see frequent references to information seen or taken from “the internet” being discussed which provides evidence of geographically diverse engagement and the exploitation of material for ever-proliferating conspiracy theories.

Similarly, Caiani and Kroll (2015) who focused on the internationalization of the extreme right and found that the internet was used to attract new members with appealing websites and interactive elements such as survey’s, chats, and forums. This research found that the Klan utilized Web 2.0 technology to propagate new ideas among like-minded people and connect individuals and organizations by creating cyber-communities that transcend national borders. There was a clear evolution in the aesthetic of the websites, in the 1990s, the Hypertext Mark-Up Language (HTML; the early language for encoding simple document semantics), was centered on describing the documents—paragraphs, lists, fonts, titles—taking basic textual information and rendering it in a way that looks pleasing for the audience. In the early 2000s, we see the use of more precise style specifications and the creation of more design effects, including blinking text, crosses that burn, Confederate battle flags that fly in the background, and southern music that plays as you scroll. This progression from Web 1.0 to Web 2.0 technology helped transform the Klan into a community that catered to the wants and needs of different members.

The ability to engage with technological advancements is dependent on the skills and experience available. It is here that the technological capacity, organizational relations, and overlap discussed in previous sections come together. For example, the *Imperial Christian Knights of the Ku Klux Klan* state on their homepage “*Before I go further into building this website, I want to thank the WEBMASTER of United White Knights of the Ku Klux Klan for his advice and help with this website. This just shows how the Klan alliances, will help one another, even though I don’t belong to his Klan, they will grant assistance*.” From this, we see the practical need for relationships between groups and sites for material and expertise. It also points to the growing importance of webmasters and discussions of the website itself. What is particularly interesting is how some foreground and promote the websites, while others attempt to downplay their significance, or, more accurately, insist that it is not the focus or a replacement for offline activity and activism. Although, in most cases, it is a combination or changing discourse about it and prioritization. On its homepage, *The White Knights of the Ku Klux Klan* state “*This Website is very simple, we do not need lots of money for websites for looks, when money can go elsewhere to further our goals*.” This feeds into a broader trend, as many of the websites analyzed state clearly that they are not an “Internet Klan.” For example, when answering the question “*What is so traditional about the Brotherhood of Klans Knights of the KKK?*” the group responds in respect to technology and wider activities: “*We are not an internet only Ku Klux Klan. We expect our members to be active by distributing literature, attending protests, private events etc*.,” thus returning us to both the earlier discussion of Klan and wider movement organizational relations and the issue of outreach activities, which also utilize and depend on technology even when they are offline.

In addition to hyperlinks and other issues already mentioned, technology is also intertwined with Klan outreach tactics in a range of ways, including the presence of contact email addresses, access to open and closed communication, discussion forums, membership forms, and donation stores. The latter is of particular importance, as efforts by organizations and activists to raise funds for their activities via the internet are longstanding. Twelve of the twenty-six websites had financing methods specifically for merchandise and were complete with translation and currency converters to help broaden markets. More specifically, the data highlights that the option to submit donations and pay for goods has transformed with advancements in technology, from Web 1.0 to Web3. Most notably, *The Knights Party* evolved from asking customers or donators to pay via cash and checks to PO boxes, to the use of credit and debit cards, and, as of 2017, payment via the digital cryptocurrency Bitcoin—quasi-private technology that allows for one level of disconnect to aid anonymity. Importantly, the Klan’s adoption of Blockchain—a type of distributed ledger technology (protocols that manage data storage distributed and replicated across multiple facilities with different owners) for recording data about transactions and/or ownership of assets in successive accumulating blocks which are shared by a network and cannot be changed by individual participants—demonstrates that although the Klan is typically not associated with cutting-edge technology, certain websites have kept up with the technological advancements to suit their purpose. This analysis has thus provided clear evidence that as new forms of online communication emerge, the Klan continually adopts it, demonstrating technological adaptability. Having examined the organization, technology and outreach, we will now return to the Klan’s central focus and rationale, that of whiteness and racism.

## Whiteness and Racism

As previously noted, each era of the Klan emerged in response to complex interactions between political developments and the Klan’s construction of threats to whiteness, the nation, and Christianity. In each era, the Klan used this matrix to reconstitute and rearticulate a perceived “crisis” for white Americans and the US in contemporary terms as a basis for recruiting members and mobilizing activists. For example, in the fifth-era, as the far right moved online, we saw a transition from what we term “TradKlan”—a traditional system supportive, possessive and defensive white supremacy and white Christian nationalism—to a more radical and insurgent, even fascist and anti-government one. The Web changed both the way the far right communicated and how they constructed and mobilized around whiteness, including how they used the internet to construct a unified, global, white identity and allowed particular narratives to develop and collect evidence across time and geographical space (Back et al., 1998; Perry & Scrivens, 2016; Winter, 2019).

In the data, terminology related to whiteness was a significant theme that emerged, which may be unsurprising for a white supremacist organization. What is unique, is how it changes over the period to reflect social and political conditions and strategic needs, as well as how seemingly contradictory discourses and narratives about whiteness co-exist within and across sites. One of the dominant ways that whiteness is constructed is through claims and narratives of white victimization, persecution, and loss of power in America, and globally. The victims of alleged reverse racism, Jewish conspiracies, hate crime, minoritization, and replacement. This is something that emerged in the fifth-era and has become mainstream in the current reactionary “culture war” across the west and global north.

Differences in ways that whiteness, and this predicament, is constructed and represented can be in terms of local, regional, or national identities or contexts in the US or globally, as well as across groups. It can even be a feature of mobilization and marketing strategies of the individual Klan groups. As noted earlier, the *Brotherhood of Klans* foreground not American whiteness, but the “*ideas and ideals of Western Christian Civilisation*.” Moreover, it is open to other pro-white organizations and activists that share their values, represented in the notions of the “*unselfish desire to secure the existence of our race and a future for white children*.” This is one of two references to David Lane’s “14 words,” one of the original white genocide claims dating to the mid-1980s fifth-era-Klan. We also see the threat to white people and activists nationally and globally on their website in examples ranging from news reports that “*illegal aliens murder 12 Americans daily*” and the murders of Eugene Terre Blanche and other “*WHITE Afrikaner farmers*” by Black people in South Africa, a rallying cry for the American far right, including Aryan Nations, in the post-Apartheid era.

The *Knights Riders Knights of the Ku Klux Klan* website is less global in their focus on whiteness and return to a more “TradKlan” system of support, or loyal constructions of whiteness as American and Christian, stating on its homepage “*Wake up White America*.” According to one member’s post, “*most people are unaware that millions of white people have been victims of violent hate crimes, especially in the cities where poor white families have been unable to escape*” and another claims “*more than 1,600 whites are murdered by Blacks each year. Blacks murder whites at 18 times the rate whites murder Blacks*.” We also see a series of local and state news stories detailing alleged anti-white hate crimes and white victimization at the hands of criminals. These include articles with the following headlines: “*Random Black-on-White stabbings in Beverley Hills leaves one dead*”; “*Hispanic Beats 12 year old White Boy – busts spleen*” and “*Non-White Kills White man with Baseball Bat*.” In the latter, the white man in question is a Russian immigrant so, despite the overwhelming focus on immigration as “the problem” for the Klan, this shows race is the issue and whiteness is the priority. In order to mitigate that and construct a form of reverse racism, the site uses a two-pronged strategy of criticism and disavowal. For example, in 2020 it openly criticized Black Lives Matter (BLM) for prioritizing Black lives, while earlier in 2015, it denounced the Charleston shooter. This enables the Klan to admonish the mainstream, stating “*Will the media be courageous enough to report this denouncement or will their silence show a hidden agenda?*” It is a strategy that allows them to represent white people and the “white resistance” as the ultimate victims of white supremacist terror attacks and anti-racist sentiment. Despite this current narration of reverse racism in response to BLM, the Klan also narrates supposed support for Black lives. Here the narrative is constructed retrospectively in their history as they claim to have “*accepted and even recruited Blacks*” and that the “*Ku Klux Klan was formed on behalf of people that wanted a decent living, both black and white*.”

The data also highlights the transnational nature of the Klan online. News stories are integrated from other countries identified as “white.” Examples include news articles referencing anti-white sentiment from the United Kingdom with headlines such as “*Racism Row Over Fan’s Flag*” (St George’s flag), and a story hailing a British tourist for refusing to “*Give up Rosa Parks’ Tribute Seat*.” Additionally, a larger global picture—drawing on the *Brotherhood of Klan’s* civilizational identification—emerges in the sites’ reproduction of Pat Buchanan’s “Death of the West” writings and rhetoric immediately following the terrorist attacks of September 11. This is housed alongside articles citing immigration as leading to the minoritization of whites in major American cities, including Pittsburgh, which is cited as being “*only 67.63% white according to the 2000 census*.” Stories about anti-white crime, white victimization, a reversal of racial power, and potential replacement are thus connected in the larger narrative that emerges, showcasing the way these strands are woven together in their contemporary articulation. This narrative strategy also maintains older threads constructing threats from prior eras. In the 2008 article “*Obama LOVED by Communist Party, USA, Don’t Forget the 22 Million White Christians Killed in the Marxist/Communist Revolution of Eastern Europe*,” we see a white victimization narrative articulated through cold war, cultural Marxism, fifth-era ZOG, and more recent white genocide or white replacement ones. This also unites the national and the global, and points to the group’s relationship to the state and government. The latter of which has always been central and contingent from the Confederacy and states’ rights in the first and third eras to 100% Americanism in the second and anti-government patriotism in the fifth. While the Knights Party attribute government responsibility for many social ills and things they allege, oppose, and think have destroyed America, including crime, terrorism, immigration, sexual deviance, reverse racism, and white victimization, they still express a possessive and defensive nationalist and patriotic message.

## Conclusion

With this article, we have demonstrated that neither traditional organizations like the Klan, nor organizational websites have gone away, but have diversified and evolved. We have drawn upon and added to existing studies that have looked at the communication, community formation, and political outreach of the Klan and wider far right to show how the use of networked technology has enabled the Klan to grow beyond their traditional regional or national boundaries and connect internationally, adapt to changing needs, conditions and opportunities, as well as organize and mobilize offline. By obtaining a sample for each of the major online Klan groups, this paper has provided clear evidence that as new forms of communication have emerged, the Klan has adopted it to not only engage in outreach and communicate, but strategize, and raise funds through merchandising sales. Although there are limits to their technological adaptation. What is most unique and paradoxical about the Klan, as the oldest, most well established and iconic far-right organization, is the way they have adapted to new conditions, political demands, and technologies throughout their history, including as early adapters of the Web and websites, they largely rested on this as others have moved on to more advanced technologies. We believe that the history of the Klans’ use of websites is testament to their commitment to this technology and “tradition,” as well as resistance to technological trends that have seen the far right mainstream, but also fragment. Our focus on this and our findings serve to challenge the presentism of the research and counterextremism field and the assumption that the use of newer, more technologically advances platforms (which themselves are sometimes short lived unlike Klan websites) ought to be a focus to understand the threat.

This research also adds value and insight to comparable work by offering a unique historical insight into the evolution of Klan websites, detailing how they have made use of Web 1.0, Web 2.0, and Web3 technologies, and the way that interacts with their ideology and strategies. Our findings, although focused on the Klan and the US, are important internationally for several reasons. Firstly, as noted, the Klan is the largest and most established far-right organization historically, and the longest active, thus providing us with the most longitudinal data to assess longer term websites and online communication in a way that allows for the mapping of continuities, discontinuities, change and comparison. This knowledge can then be applied to other studies and context. Secondly, as highlighted in the introduction and survey of research themes (identify formation, recruitment, radicalization), and in the study itself (history, merchandising, race), what we are looking at is relevant to wider research and interest in digital hate, far right movements and online communication in different regional, national, and global contexts. This knowledge can help us understand how the Klan and similar organizations may adopt and use future technological innovations, or resist them, and thereby how we can best pick up on and counter online extremist activity, that may, on the outset, appear hidden. The embracing of new waves of communication technology also suggest a regular influx of new and younger members across each generation, which, in turn, provides useful information to help understand the diverse and changing nature and character of the far right and how it operates, communicates, competes, and mobilizes. Thirdly, theoretically, we show the theorization of digital hate and online communication is limited by technological and platform presentism, and a focus on social media and radicalization. As such, we argue that a long-term analysis of continuity and change on alternative and traditional platforms can help add to the wider theorization of online hate, the development of far-right discourses, identities, practices, strategies, and organizations. This occurs throughout the paper, but specifically in the analysis and insights provided by the data. In terms of its practical and policy uses, the research and analysis should encourage researchers, policy makers and stakeholders to pay greater attending to historical movements, historical change, different approaches to technology and communication and multi-tasking organizations, and a wider variety of operational logics than just the newest and most high profile. The longitudinal approach to data collection and analysis, which within far right research is only really possible by looking at Klan websites, has shown while there is less reliance on websites as there once were, there is more diversity in approaches, and they remain an important component of the contemporary far-right identity and community building, as well as the wider online far-right extremist and terrorist ecosystem.

## Notes

1. Linguistic inquiry and word count is a text analysis tool that allows users to extract social and psychological insights from written texts by reading every word, number and piece of punctuation in a given text and comparing this against a pre-defined dictionary; the results for each category in the dictionary are then output as a percentage of total words.
